# Prevalence of Vitamin D Deficiency Among Healthy Young Adults at Kuwait University

**DOI:** 10.7759/cureus.75911

**Published:** 2024-12-17

**Authors:** Mohammed A Jamali, Suad M Abdeen, Thazhumpal C Mathew

**Affiliations:** 1 Department of Psychiatry, Faculty of Medicine, Health Sciences Center, Kuwait University, Kuwait City, KWT; 2 Department of Pathology, Faculty of Medicine, Health Sciences Center, Kuwait University, Kuwait City, KWT; 3 Department of Medical Laboratory Sciences, Faculty of Allied Health Sciences, Kuwait University, Kuwait City, KWT

**Keywords:** 25-hydroxyvitamin d, 25(oh)d, calcitriol, hypovitaminosis d, vitamin d3, vitamin d deficiency, vitamin d insufficiency

## Abstract

Objective: This study aims to investigate the prevalence of vitamin D deficiency among healthy young medical and dental students at Kuwait University.

Methods: This cross-sectional study included a total of 201 medical and dental students (male = 99; female = 102) at Kuwait University. Blood samples were collected to assess 25-hydroxyvitamin D (25(OH)D) by electrochemiluminescence (ECL) immunoassay, and a questionnaire was distributed to address related qualitative data. P-values less than 0.05 were considered significant.

Results: Vitamin D deficiency (<50 nmol/L) was reported in 171 (85.1%) of the participants. A total of 17 (8.5%) participants exhibited insufficient vitamin D (50.1-75 nmol/L), and only 13 (6.5%) students had optimal vitamin D (>75 nmol/L). According to gender, vitamin D deficiency was more common in male students (89, 89.9%) compared to females (82, 80.4%). Vitamin D levels for students in the clinical academic years (sixth and fifth years) were significantly higher (P < 0.001) compared to the non-clinical years (fourth, third, and second years).

Conclusion: The prevalence of vitamin D deficiency was very high among medical and dental students of Kuwait University. The students with high academic years suffered more from vitamin D deficiency.

## Introduction

Vitamin D deficiency remains high worldwide and is causing a negative burden on the health economy [1]. The overall global prevalence ranges from 30% to 50% [2], and 83% of adults living in Kuwait have vitamin D deficiency [3]. Vitamin D deficiency is causing high costs due to the disease burden. Therefore, achieving population vitamin D sufficiency will help to reduce the healthcare cost [1].

Vitamin D also contributes to many chronic diseases, such as depression, metabolic syndrome, and cardiovascular risks [4]. Although the pathophysiology is still unclear, vitamin D deficiency is a risk factor for developing the components of metabolic syndrome and its consequences of diabetes and cardiovascular disease, increasing morbidity and mortality [4]. Vitamin D deficiency is also a well-documented contributing factor for depression [5], cancer [6], and chronic diseases, including multiple sclerosis [7] and other autoimmune diseases [8].

Cui et al. (2023) conducted a systematic review and meta-analysis to determine global and regional prevalence between 2000 and 2022 among 7.9 million participants from 308 included studies and 81 different countries. The estimation showed that 63.6% of the population showed vitamin D deficiency, and 76·6% showed vitamin D insufficiency. The prevalence has slightly decreased in the first decade of the 21st century compared to the second decade. However, it has remained high for individuals, especially those living in high altitude levels, during the winter to spring seasons (1.7 times more), individuals from lower-middle-income countries, and among females. The authors emphasized that the government and policymakers should consider comprehensive public health policy steps to prevent its prevalence because it may also enhance the overall disease burden [9].

The primary circulating form of vitamin D that physicians measure in the blood to determine vitamin D status is 25-hydroxyvitamin D (25(OH)D), also called calcifediol, a prohormone produced in the liver [10]. Vitamin D refers to a group of lipid-soluble molecules that contain a four-ringed cholesterol backbone [11]. Vitamin D precursors need to undergo several steps to obtain the bioactive version of vitamin D, which is calcitriol or 1,25-dihydroxy vitamin D [12]. Vitamin D3 and D2 can both be obtained from diet and supplements; however, vitamin D3 can be synthesized nonenzymatically in the skin from previtamin D3 (7-dehydrocholesterol) in the presence of UV light from sunlight. Vitamin D3 and vitamin D2 will undergo hydroxylation in the liver into 25-hydroxyvitamin D (1,25(OH)2D) [13].

The active functional metabolite calcitriol can then enter the target cells by passive diffusion to bind to the cytosol's vitamin D receptor (VDR). Two receptors will then dimerize and subsequently travel to the hormone-responsive element (HRE) in the genomic DNA to mediate the synthesis of calcium-binding protein that will increase calcium absorption in the gut [14]. The main stimulator for calcitriol production is the parathyroid hormone (PTH), and the major inhibitor is increased serum calcium levels [15]. The most well-known function of calcitriol is to maintain calcium (Ca) and phosphate (PO_4_) within the normal physiological levels by the absorption of dietary Ca and by PO_4_ the small intestine enterocytes, reabsorption for Ca and PO_4_ from the distal renal tubules, and lastly by activating bone osteoclasts to mobilize Ca from bone to blood if levels remain low [15].

Vitamin D deficiency predictors are insufficient exposure to sunlight, dietary intake, age, gender, ethnicity, season, socioeconomic status, drinking, and lower milk consumption [16]. Approximately 50% to 90% of vitamin D is synthesized when the skin is exposed to a specific wavelength of ultraviolet B (UVB) rays (280-320 nm) from sunlight, whereas the rest is gained by diet [17]. In addition, individuals suffering from chronic kidney disease [18] and liver disease are susceptible to vitamin D deficiency because of the disturbance of the enzymatic hydroxylation step necessary to activate vitamin D [19].

Rationale of the study

The rationale behind this study is to assess and determine the prevalence of vitamin D deficiency among healthy young adults (male and female) medical and dental students of Kuwait University. However, no published studies specifically demonstrated the vitamin D status of healthy young adults in Kuwait.

The objectives of the study are to investigate the prevalence of vitamin D deficiency among healthy young medical and dental students of Kuwait University. Specifically, this study aims to determine the significance of predictors (education, gender) of vitamin D deficiency and to assess the general health characteristics (age, BMI, waist circumference, blood pressure, fasting blood sugar, lipid profile, liver function, and renal function tests) of the healthy young adults by gender.

## Materials and methods

Study design and setting

This was a cross-sectional study conducted among faculty of medical and dental students of Kuwait University.

Sampling technique

A stratified random sampling technique was used after taking a defined adult population of medical college aged between 18 and 25 years. The total number of students data was also taken to ensure equal representation of both genders. The individual strata based on gender were generated and allocated randomly using a randomization number list to avoid selection bias.

Sample size estimation

The sample size was calculated using the OpenEpi app, taking the estimated prevalence among females as high as 91.69%, i.e., p1 = 0.917, and males as 70.32%, i.e., p2 = 0.703 [20]. In order to be 80% certain (1-Beta = 0.8) to detect the prevalence ratio of RR as 0.805 using alpha levels of 0.05, recruiting both genders proposed 77 individuals in each group. The 154-sample size was proposed, but 201 were taken to increase the validity of the sample size findings.

Sample population recruitment

The study was conducted in the Faculty of Medicine of Kuwait University. The inclusion criteria for our sample population consisted of healthy medical and dental students of Kuwait University in their second, third, fourth, fifth, and sixth academic years who agreed to give a blood sample, answer the questionnaire, and sign the consent form. The students were randomly and anonymously selected using the class list, and the total planned target sample population was 200 healthy students, composed of 100 males and 100 females from each recruited group. We excluded first- and seventh-year students due to difficult accessibility. We excluded participants under 21 years old without parental consent, as well as those who agreed to do the questionnaire but did not give a blood sample and vice versa.

Consent form, questionnaire, and anthropometric parameters

The data collection started in the Pathology Department at the Faculty of Medicine, first by acquiring a signed adult's or parental consent form from each participant. The consent forms clearly explained the objectives, risks, and benefits of the project, the blood collection procedure, and the participant's rights, which include data confidentiality and the right to leave the study at any desired time. The consent form clarified that once all lab analyses related to the project are completed, all blood samples will be destroyed and will not be used for future research, as requested by the Ethical Committee. The participants then answered a three-minute questionnaire with 16 questions created based on the required information, including demographic and qualitative clinical information. A blood pressure reading was obtained after sitting and resting in a private room for 10 minutes as a standardization method; anthropometric measurements, including waist circumference, weight, and height, were obtained afterward.

Blood collection

After signing the consent form, answering the questionnaire, and measuring the anthropometric parameters, certified licensed phlebotomists from Mubarak Al-Kabeer Hospital (MKH) drew 12 cc of blood from each participant; blood samples were immediately labeled with serial numbers that matched the label on the questionnaire for each participant; this was done to protect the participant's identity and confidentiality. The samples were then immediately kept in a cold icebox for temporary storage until transferred to the MKH laboratory to be analyzed on the same day.

Biochemical analysis of serum samples

Plasma 25(OH) vitamin D concentration was measured by electrochemiluminescence (ECL) immunoassay by using a Cobas e 411 analyzer (Cobas e 411 analyzer; F. Hoffmann-La Roche AG, Basel, Switzerland). Total cholesterol (Tchol), HDL-cholesterol, triacylglycerol (TG), fasting blood sugar (FBS), urea, creatinine, aspartate aminotransferase (AST), alanine aminotransferase (ALT), free thyroxine (T4), and thyroid-stimulating hormone (TSH) were measured on an automated analyzer (DXC 800; Beckman Coulter, Brea, CA, USA). LDL-cholesterol was calculated by the following formula: $LDL = (0.97 &times; TC) - (0.93 &times; HDL) - (0.19 &times; TG)$ [21]. In addition, hemoglobin (Hb), white blood cells (WBC), and platelets were obtained using the hematology analyzer (LH 750; Beckman Coulter, Brea, CA, USA).

Statistical analysis

Data entry and analysis were completed using IBM SPSS Statistics for Windows, Version 25 (Released 2017; IBM Corp., Armonk, New York). The mean and standard deviation (±SD) were presented for continuous and count (n) and percentage for categorical variables. Vitamin D status was categorized according to the American Endocrine Society guidelines: optimal = >75 nmol/L, insufficiency = 50.1-75 nmol/L, and deficiency = <50 nmol/L [22,23]. P-values for the continuous variables were generated using the non-parametric Mann-Whitney U test and Kruskal-Wallis H test; except for HDL and Hb, an independent-sample t-test was used. Pearson's chi-square test was used to calculate P-values for categorical variables. Cramer's V test was used to establish the effect size for the chi-square test with violated assumptions. P<0.05 was considered statistically significant.

Ethical committee and funding approval

This publication is part of a Master's Degree Thesis (Thesis Identification: 0545598) funded and registered by the College of Graduate Studies of Kuwait University. The thesis is also listed in the National Library of Kuwait (ISBN: 978-99906-1-546-3). This project was ethically approved by the Joint Research Ethical Committee of the Ministry of Health of Kuwait and the Faculty of Medicine of Kuwait University. A parental consent form for minors under 21 years old and an adult consent form were reviewed and approved by the committee in both Arabic and English versions.

Efforts to report and prevent bias

The write-up was done following the Strengthening the Reporting of Observational Studies in Epidemiology (STROBE) guidelines to ensure the reproducibility and replicability of the study. The standardized methods and biochemical assay techniques were utilized to determine accurate levels and compare them with standardized statistics to ensure the validity of the findings.

## Results

The total study population sample size was 201 (99 males and 102 females). The mean age for males was 21.22 (±1.5) years versus 21.26 (±1.6) years for females. As for the anthropometric measurements in this study population, the means of BMI and waist circumference in women were found to be 23.87 (±4.42) kg/m^2^ and 74.41 (±9) cm, respectively, while in men, they were 26.86 (±4.7) kg/m^2^ and 91.84 (±14.1) cm. BMI and waist circumference are significantly different across genders (P-value < 0.001).

Blood pressure readings significantly differed across genders; the mean systolic and diastolic BP for men were 123.26 (±7.3) mmHg and 78.76 (±6.5) mmHg, respectively, whereas for females, they were found to be 113.23 (±8.4) mmHg and 74.63 (±7.1) mmHg, respectively. Moreover, the mean FBS was found to be 4.56 (±0.68) mmol/L for males and 4.3951 (±0.46) mmol/L for females, showing a statistically significant difference between males and females (P-value < 0.045). As for lipid profile, the mean Tchol was not significantly different between the male and female groups (4.6479 (±0.85) for males and 4.448 (±0.69) for females). The difference in means, however, was significant (P-value < 0.001) for TG, HDL, and LDL, as they were found to be 0.8154 (±0.44) mmol/L, 1.1729 (±0.22) mmol/L, and 3.0924 (±0.8) mmol/L in males, and 0.5378 (±0.25) mmol/L, 1.4798 (±0.3) mmol/L, and 2.7133 (±0.59) mmol/L in females. Other biochemical variables, including ALT, AST, creatinine, urea, Hb, platelet, and free T4 of males, were significantly greater (P-value < 0.001) than females. TSH did not significantly differ across genders (P-value > 0.05).

As for the distribution of this study, participants by academic year showed 41 (20.1%) from the sixth year, 33 (16.2%) from the fifth year, 45 (22.1%) from the fourth year, 42 (20.6%) from the third year, and 40 (19.6%) from the second year. Out of the total 201 participants, only one male was diagnosed with diabetes, one female was diagnosed with hypertension, and another one female was diagnosed with dyslipidemia.

None of the 102 females participating in this study reported being smokers, while among the 99 male participants, 23 (23.2%) of them were smokers. The study showed more females who do not exercise (64, 62.7%) than males (23, 23.3%). There were 38 (37.3%) female participants who exercised one to two days a week, while male participants were 76 (76.8%). None of the male or female participants in our study exercised for more than two days a week (Tables 1, 2).

**Table 1:** Background characteristics of healthy young adults by gender *P-values were generated using the non-parametric Mann-Whitney U test. **Except for HDL and Hb, an independent-sample t-test was used. Shapiro-Wilk test was used to assess normality (not shown). A P-value < 0.05 was considered statistically significant. BMI: Body Mass Index; WC: Waist Circumference; BP: Blood Pressure; FBS: Fasting Blood Sugar; Tchol: Total Cholesterol; TG: Triglycerides; HDL: High-Density Lipoproteins; LDL: Low-Density Lipoproteins; ALT: Alanine aminotransferase; AST: Aspartate transaminase; WBC: White Blood Cells; Hb: Hemoglobin; TSH: Thyroid-Stimulating Hormone; Free T4: Free Thyroxine

Criteria	Females (N = 102)	Males (N = 99)	P-value	Test Statistics
	Mean (±SD)	Mean (±SD)		
Age (years)	21 (±2)	21 (±2)	0.827	5137.50*
BMI (kg/m^2^)	23.88 (±4.43)	26.87 (±4.7)	<0.001	7087.50*
WC (cm)	74.41 (±9)	91.85 (±14.1)	<0.001	8736.50*
Systolic BP (mmHg)	113 (±8)	123 (±7)	<0.001	8206.00*
Diastolic BP (mmHg)	75 (±7)	79 (±7)	<0.001	6580.00*
FBS (mmol/L)	4.4 (±0.46)	4.57 (±0.68)	0.045	5873.00*
Tchol (mmol/L)	4.45 (±0.69)	4.65 (±0.85)	0.076	5780.50*
TG (mmol/L)	0.54 (±0.26)	0.82 (±0.45)	<0.001	6928.50*
HDL (mmol/L)	1.48 (±0.31)	1.17 (±0.23)	<0.001	**8.00
LDL (mmol/L)	2.71 (±0.60)	3.09 (±0.8)	<0.001	6299.00*
ALT (U/L)	15.71 (±7.08)	29.67 (±15.16)	<0.001	8711.00*
AST (U/L)	18.9 (±3.52)	24.66 (±9.92)	<0.001	7354.50*
Creatinine (μmol/L)	49.79 (±7.88)	75.74 (±13.07)	<0.001	9713.50*
Urea (mmol/L)	3.14 (±0.96)	4.27 (±1.12)	<0.001	7852.00*
WBC K/µL)	7.18 (±1.73)	6.84 (±1.7)	0.194	4422.50*
Hb (g/L)	131.01 (±9)	155.30 (±10.09)	<0.001	**-17.91
Platelet (K/µL)	273.66 (±51.29)	238.54 (±49.38)	<0.001	2871.00*
TSH (mIU/L)	1.69 (±1.2)	1.86 (±0.94)	0.081	5768.50*
Free T4 (μg/dL)	11.04 (±1.1)	11.62 (±1.48)	<0.001	6333.50*

**Table 2:** Background characteristics of healthy young adults by gender The chi-square test was used to generate P-values to test the difference between male and female participants. A P-value < 0.05 was considered statistically significant.

Criteria, n (%)	Total (N = 201)	Female (N = 102)	Male (N=99)	Chi-Square (P-value)
Academic year	-	-	-	-
6th year	41 (20.1%)	20 (20.2%)	21 (20.6%)	0.375 (0.984)
5th year	33 (16.2%)	15 (15.2%)	18 (17.6%)	
4th year	45 (22.1%)	22 (22.2%)	23 (22.5%)	
3rd year	42 (20.6%)	21 (21.2%)	21 (20.6%)	
2nd year	40 (19.6%)	21 (21.2%)	19 (18.6%)	
Diagnosed with diabetes	-	-	-	-
Yes	1 (0.5%)	0 (0%)	1 (1%)	1.035 (0.309)
No	200 (99.5%)	102 (100%)	98 (99%)	
Diagnosed with hypertension	-	-	-	-
Yes	1 (0.5%)	1 (1%)	0 (0%)	0.975 (0.323)
No	200 (99.5%)	101 (99%)	99 (100%)	
Diagnosed with dyslipidemia	-	-	-	-
Yes	1 (0.5%)	1 (1%)	0 (0%)	0.975 (0.323)
No	200 (99.5%)	101 (99%)	99 (100%)	
Smoking	-	-	-	-
Yes	23 (11.4%)	0 (0%)	23 (23.2%)	26.76 (<0.001)
No	178 (88.6%)	102 (100%)	76 (76.8%)	
Exercise	-	-	-	-
No exercise	87 (43.3%)	64 (62.7%)	23 (23.2%)	31.951 (<0.001)
1-2 days per week	114 (56.7%)	38 (37.3%)	76 (76.8%)	
3-5 days per week	0 (0%)	0 (0%)	0 (0%)	

The mean vitamin D level was 34.28 (±28.9) nmol/L for males and 34.55 (±13.2) nmol/L for females, with no significant difference found between the two groups. As shown in Table 3, the total number of individuals in this study sample group with vitamin D deficiency (<50 nmol/L) was 171 (85.1%). The individuals with insufficient vitamin D were found to be 17 (8.5%). Only 13 (6.5%) individuals were found to have optimal levels of serum vitamin D. Gender comparison showed that vitamin D deficiency was more common in males (89, 89.9%) compared to females (82, 80.4%). Vitamin D insufficiency and optimal levels in males were found to be seven (7.1%) and three (3%), respectively, compared to females, where it was 10 (9.8%) for both. Out of 201 participants, 33 (16.4%) have never undergone a routine blood test, 141 (70.1%) have never done a vitamin D blood test, and 164 (80.4%) have never received vitamin D supplementations. Table 3 shows males to be less adherent to blood testing and vitamin D supplementations.

**Table 3:** Descriptive analysis of vitamin D qualitative and quantitative variables *P-values were generated using the chi-square test. **Except for vitamin D levels, the P-value was calculated using the non-parametric Mann-Whitney U test. ^One of the assumptions of the chi-square test was violated, but Cramer's V was then run to establish the effect size, and the relationship was found to be significant at P < 0.001. Shapiro-Wilk test was used to assess normality (not shown). A P-value < 0.05 was considered statistically significant.

Criteria	Total (N = 201)	Male (N = 99)	Female (N = 102)	P-value	Test Statistics
Vitamin D levels (nmol/L), mean (±SD)	34.42 (±22.55)	34.56 (±13.29)	34.28 (±28.92)	<0.001	6682.00**
Vitamin D status, n (%)	-	-	-	-	-
Deficiency	171 (85.1%)	89 (89.9%)	82 (80.4%)	0.103	4.541*
Insufficiency	17 (8.5%)	7 (7.1%)	10 (9.8%)		
Optimal	13 (6.5%)	3 (3%)	10 (9.8%)		
Last routine blood test, n (%)	-	-	-	-	-
Never	33 (16.4%)	19 (19.2%)	14 (13.7%)	0.111	6.012*
<6 months	85 (42.3%)	36 (36.4%)	49 (48%)		
6 months to 2 years	47 (23.4%)	21 (21.2%)	26 (25.5%)		
>2 years	36 (17.9%)	23 (23.2%)	13 (12.7%)		
Last vitamin D blood test, n (%)	-	-	-	-	-
Never	141 (70.1%)	83 (83.8%)	58 (56.9%)	<0.001	22.580*
<6 months	26 (12.9%)	11 (11.1%)	15 (14.7%)		
6 months to 2 years	28 (13.9%)	5 (5.1%)	23 (22.5%)		
>2 years	6 (3%)	0 (0%)	6 (5.9%)		
Vitamin D supplements, n (%)	-	-	-	-	-
Never taken	164 (80.4%)	92 (92.9%)	72 (70.6%)	<0.001^	18.708*
Previously taken	24 (11.8%)	3 (3%)	21 (20.6%)		
Currently taking	7 (3.4%)	3 (3%)	4 (3.9%)		
Both previously and currently	6 (2.9%)	1 (1%)	5 (4.9%)		

**Figure 1:**
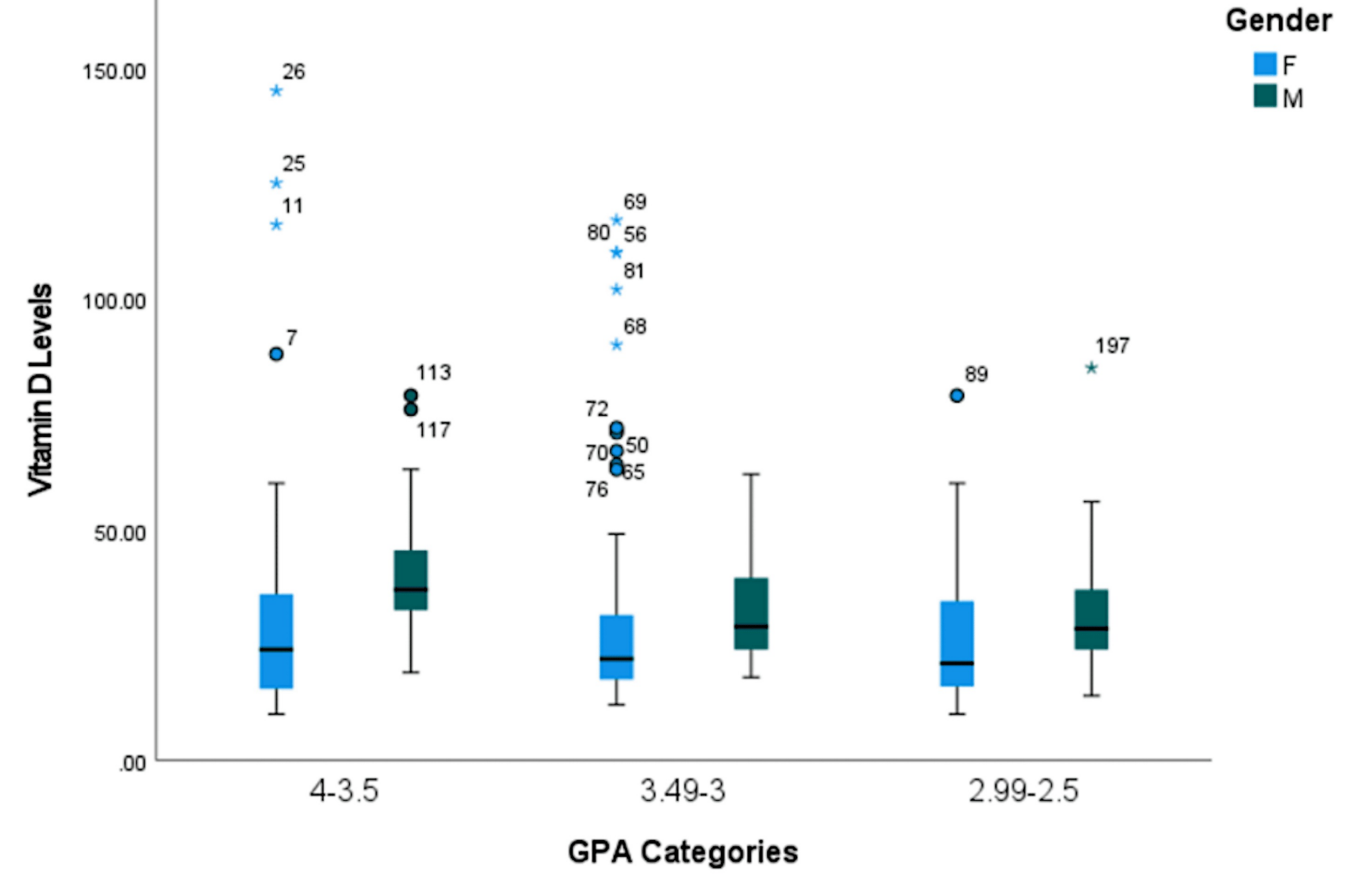
Boxplot demonstrating the distribution of vitamin D levels (nmol/L) over GPA categories (4-3.5, 3.49-3, and 2.99-2.5), stratified by gender with unadjusted P-value.

The box plot illustrates that male participants have higher median vitamin D levels among all GPA categories compared to female participants. There is also reporting of widespread vitamin D levels, particularly in higher GPA categories 4-3.5 and 3.49-3 (Figure 1).

The male students with higher academic performance had significantly higher vitamin levels (P = 0.003). The male students with GPA = 4-3.5 had 39.97 (±14.1), whereas students with GPA = 3.49-3 had 32.71 (±10.86), and GPA = 2.99-2.5 had 28.9 (±8.95). The female students did not show this significant association (P > 0.05). Vitamin D levels differed significantly across the academic year for both genders (P < 0.001). The vitamin D levels for the total participants in the clinical academic years (sixth and fifth years) were higher compared to the non-clinical years (fourth, third, and second years). The participants in the sixth and fifth years had 39.8 (±23.86) and 43.36 (±31.73), respectively, compared to 25.2 (±13.57), 31.29 (±22.49), and 35.18 (±15.91) for the fourth, third, and second academic years (Table 4).

**Table 4:** The effect of academic performance and academic year on vitamin D levels according to gender The P-value was generated using the non-parametric Kruskal-Wallis H test. Shapiro-Wilk test was used to assess normality (not shown). A P-value < 0.05 was considered statistically significant. GPA: Grade Point Mean

Parameter	Vitamin D levels (nmol/L)		
	Total (N = 192)	Male (N = 94)	Female (N = 98)
Academic performance (measured by 4-point GPA scale)	-	-	-
4–3.5	38.52 (±26.09)	39.97 (±14.1)	37.06 (±34.35)
3.49–3	33.7 (±23.38)	32.71 (±10.86)	34.29 (±28.52)
2.99–2.5	29.07 (±13.18)	28.9 (±8.95)	29.4 (±19.67)
Kruskal-Wallis H test (P-value)	5.066 (0.471)	9.424 (0.003)	0.252 (0.882)
-	Vitamin D levels (nmol/L)		
-	Total (N = 201)	Male (N = 99)	Female (N = 102)
Academic year	-	-	-
6th year	39.8 (±23.86)	40.85 (±14.09)	38.81 (±30.78)
5th year	43.36 (±31.73)	33.53 (±15.72)	51.56 (± 39.18)
4th year	25.2 (±13.57)	27.32 (±8.76)	23.17 (±16.92)
3rd year	31.29 (±22.49)	34.71 (±13.68)	27.86 (±28.72)
2nd year	35.18 (±15.91)	36.71 (±11.55)	33.47 (±19.85)
Kruskal-Wallis H test (P-value)	18.890 (<0.001)	8.873 (0.004)	17.790 (<0.001)

## Discussion

The issue of vitamin D deficiency was raised a few decades ago and remains unsolved. Surprisingly, more recent studies still demonstrated a high prevalence of vitamin D deficiency among developed and undeveloped countries. It has been a significant contributing factor to many chronic physical, mental, and medical conditions [24].

Although WHO declared vitamin D a global health concern [25], particularly since the pandemic of COVID-19 in March 2020, the response of global and local countries toward vitamin D deficiency, such as updating food fortification and guidelines, was not optimal. However, this response was limited due to evolving societal norms, lifestyle modifications, and social media. Moreover, vitamin D deficiency is reported to be a risk factor for many chronic diseases, such as depression [5,8,18]. Many articles published in the past decades have emphasized awareness campaigns and evidence-based guidelines for the prevention of vitamin D deficiency. However, vitamin D deficiency persists at very concerning levels [26,27].

Having a suitable geographical location and an adequate amount of sunlight exposure, it is widely expected that the Kuwaiti population should have optimal vitamin D levels [28]. However, this cross-sectional study showed vitamin D deficiency in 171 (85.1%) among 201 randomly selected medical and dental students (44.2% male, 40.7% female). Students with vitamin D insufficiency were 8.5%, while only 6.5% had optimal vitamin D levels.

Similar studies in other subpopulations in Kuwait also showed a high prevalence of vitamin D deficiency: 10-60% in mothers and 40-60% in their newborns at delivery [29], 25% in preschool children [30], 60% in type-1 diabetic (T1D) children [31], 80.2% in adolescents [32], 64% in male athletes [33], and 63% among older adults [34]. However, no published studies specifically demonstrated the vitamin D status of healthy young adults in Kuwait. Furthermore, countries within the same geographic area demonstrated close observations. A cross-sectional study published in 2019 for Qatar comprised 102,342 participants (adults between 18 and 65 years old) and revealed 71.4% vitamin D deficiency [35]. In addition, a case-control study of 12,346 Emiratis published in 2023 showed vitamin D deficiency among 72% of participants [36]. In Saudi Arabia, 66.5% vitamin D deficiency was found among 1,093 healthy adult males with a mean age of 50.7 ± 13.9 years [37].

Globally, the prevalence of vitamin D deficiency widely varies, such as 83% in China [38], 41.3% in Europe [9], and 12.8% among USA adults [9]. A meta-analysis reported 9% vitamin D deficiency in African regions [9], whereas the prevalence of vitamin D was estimated at 28% among the Brazilian population [39]. A national survey in Australia reported 20% vitamin D deficiency [40].

The results of this cross-sectional study showed that a higher academic performance (higher GPA) was significantly associated (P-value = 0.003) with higher vitamin D levels among male students. Male students with a GPA of 4-3.5 showed mean vitamin D levels of 39.97±14.1, whereas students with a GPA of 3.49-3 and 2.99-2.5 showed lower mean vitamin D levels of 32.71 ±10.86 and 28.9 ±8.95, respectively. The vitamin D levels for all participants in the clinical academic years (sixth and fifth years) were higher than the non-clinical years (fourth, third, and second years). This finding of having lower vitamin D levels among individuals with fewer academic achievements is consistent with a study conducted in Saudi Arabia, which showed that adults with less academic education had significantly more vitamin D deficiency [41]. Another study reported that young adults with bachelor's degrees had more vitamin D deficiency than holders of master's degrees [42].

Moreover, Gamage et al. (2021) from Australia found that parents with more education and awareness from school programs encouraged children to spend more time in the sun, thereby seeking natural sources of vitamin D [43]. Chen et al. (2021) found that pregnant women with higher education levels are a protective factor. It indicates pregnant women with lower education levels also had lower 25(OH) vitamin D levels and a lower birth weight of newborns (small gestational age) [44]. On the other hand, Richard et al. (2020) did not find education a predictor of vitamin D level deficiency in pregnant women of the Democratic Republic of the Congo, and this was attributed to dark skin color [45].

The published existing literature showed that healthcare workers are widely suffering from vitamin D deficiency, raising an alarm to emphasize the need to raise awareness among future healthcare educators and providers regarding the consequences of vitamin D deficiency besides raising awareness within the public. A systemic review of 71 peer-reviewed articles, published in 2017, showed vitamin D deficiency among healthcare workers as 72% in healthcare students, 65% in medical residents, 46% in practicing physicians, and 43% in nurses [46]. As the global pandemic of vitamin D deficiency encourages healthy young adults to increase exposure to sunlight and consume food fortified with vitamin D according to evidence-based guidelines, adequate sun exposure and adequate food fortification should be considered to maintain normal physiological levels of circulating blood vitamin D3 [47].

This study found high levels of vitamin D deficiency among medical and dental students in their pre-clinical and clinical academic years. The above-discussed studies also show vitamin D deficiency in medical students, residents, and doctors in different regions of the world, demonstrating a gap in medical school curriculums that does not correspond dynamically with the global health concerns of the WHO. The upcoming generation of health services providers with minimum attention to self-awareness of vitamin D deficiency may reflect on their practice. These healthcare providers are expected to promote vitamin D awareness to their patients through clinics or health campaigns, especially with the presence of social media as a helpful tool compared to the past decades. Similarly, Zgliczyński et al. (2021) concluded in their cohort study that medical doctors need more training and education on vitamin D supplementation to better address its deficiency-related factors among the Polish population [48].

Based on the findings, the authors of this study suggest focusing on the curriculum of medical education and universities to dynamically integrate global WHO concerns into the curriculum instead of focusing on pure science. New medical students are skilled and know how to use social or digital media effectively to raise awareness among the population. They could contribute to awareness through clinics after graduation and during their studies through institutionally organized health awareness campaigns using digital media platforms. They will be able to deliver awareness to reduce the burden of vitamin D-related health issues at societal, regional, and global levels, similar to awareness programs such as Prostate Cancer Day and the HPV Awareness Program.

The study has some limitations, including sampling bias, as it is limited to medical and dental students. Future researchers are recommended to include other fields beyond medical and dental disciplines to ensure diversity in sample size. Additionally, the study design is cross-sectional, which is the limitation to establishing a causal relationship between vitamin D deficiency and academic performance. There might be chances of bias due to self-reported data, e.g., lifestyle habits, which may introduce recall bias. Furthermore, the study is only based on one single center. Therefore, the authors recommend researchers include other universities to enhance the generalizability of the findings for Kuwaiti population students.

## Conclusions

Vitamin D deficiency is alarmingly high among otherwise healthy young individuals in the Kuwaiti population, such as the medical students in our study group, necessitating urgent awareness campaigns to disseminate information on impending health problems and recommend corrective action. One of the limitations of this study is its inherent bias, which includes sampling bias, self-reported bias, and monocentric study. Future researchers are recommended to include a diverse population, such as students from other disciplines; involving students from different universities may further enhance the generalizability of the findings and ensure validity.
